# Relationship between exposure to Extremely Low‐Frequency (ELF) magnetic field and the level of some reproductive hormones among power plant workers

**DOI:** 10.1002/1348-9585.12173

**Published:** 2020-10-19

**Authors:** Sheari Suri, Somayeh F. Dehghan, Ali S. Sahlabadi, Soheila K. Ardakani, Nariman Moradi, Maryam Rahmati, Fahimeh R. Tehrani

**Affiliations:** ^1^ Department of Occupational Health and Safety School of Public Health and Safety Shahid Beheshti University of Medical Sciences Tehran Iran; ^2^ Environmental and Occupational Hazards Control Research Center School of Public Health and Safety Shahid Beheshti University of Medical Sciences Tehran Iran; ^3^ Department of Occupational Health and Safety School of Public Health and Safety Shahid Beheshti University of Medical Sciences Tehran Iran; ^4^ Department of Biostatistics School of Allied Medical Sciences Shahid Beheshti University of Medical Sciences Tehran Iran; ^5^ Cellular and Molecular Research Center Research Institute for Health Development Kurdistan University of Medical Sciences Sanandaj Iran; ^6^ Department of Clinical Biochemistry Faculty of Medicine Iran University of Medical Sciences Tehran Iran; ^7^ Department of Epidemiology and Biostatistics School of Public Health Tehran University of Medical Sciences Tehran Iran; ^8^ Obstetrics and Gynecology Reproductive Endocrinology Research Center Research Institute for Endocrine Sciences Shahid Beheshti University of Medical Sciences Tehran Iran

**Keywords:** extremely low‐frequency magnetic field, men's reproductive hormones, occupational exposure, power plant

## Abstract

**Background and Aims:**

Today, human beings are exposed to the ELF magnetic field of electrical equipment and power lines, which can damage Leydig cells and alter the secretion of reproductive hormones. The purpose of this study was to investigate the relationship between exposure to ELF magnetic field and the level of some reproductive hormones in male power plant workers.

**Materials and Methods:**

The present cross‐sectional study was carried out among all male employees of different units of the selected power plant around Tehran, Iran. All participants were asked to complete demographic data sheets and General Health questionnaire, on condition of consent and meeting the inclusion criteria. Time‐weighted average (TWA) exposure to magnetic field of 122 men was measured by IEEE Std C95.3.1 method using TES 1393 Gauss meter. Based on the exposure level, subjects were divided into three groups. Serum Levels of Free Testosterone, Luteinizing Hormone (LH), and Follicle stimulating hormone (FSH) in participants were determined. Data analysis was performed using ANOVA, Kruskal‐Wallis tests, and the relationships between variables were assessed by linear regression and correlation using SPSS v.25 software.

**Results:**

There was no significant statistical correlation between the level of ELF exposure and serum levels of free testosterone, LH, and FSH, (*r* = 0.158). Serum levels of LH decreased significantly with age and duration of work experience (*P* < .05, *r* = −.25, *P* = .005, *r* = −.203, *P* = .025).

**Conclusion:**

There was no relationship between exposure to magnetic field in power plants and reproductive hormone levels, although it is impossible to make definitive comments without using more accurate methods to estimate male fertility.

## 1 INTRODUCTION

Increasing exposure of humans to electromagnetic fields (EMFs) caused by electrical products^1^, ^2^ and exposure to extremely low‐frequency electromagnetic fields (ELFs) at all stages of production up to the consumption of electrical energy lead to adverse health effects.^3^ The amount of exposure to electromagnetic fields depends on the location, size, and distance of the user from the source.^4^ In a report, World Health Organization (WHO) announced that the exposure rate of most studied subjects to ELF magnetic field was less than 0.1 μT and a small percentage was higher than 0.3μT.^5^ In recent years, much attention has been given to the potential adverse effects of electromagnetic fields on the health of employees of high‐voltage power distribution lines, electrical substations, and power plants.^6^ The result of previous studies have shown that EMF can have harmful effects on the human body.^7^ The results of these studies included the possibility of cancer in children living near power lines,^8^ brain tumors, infertility, congenital defects, abortion, and others.^9^, ^10^, ^11^, ^12^, ^13^ According to the results of epidemiological studies, the International Agency for Research on Cancer (IARC) has classified ELF‐EMF as human carcinogen 2B.^14^ The results of some studies indicate a possible relationship between exposure to low‐frequency electromagnetic fields (LF‐EMF) and the toxic effects on reproduction and changes in the male reproductive system.^15^, ^16^ Given the increasing prevalence of infertility and issues related to sperm quality decline and the impact of environmental hazards on the male reproductive system, the potential risk of EMF in male reproductive changes and often hormonal changes has been considered.^11^ The effect of EMF radiation on reproductive performance depends on frequency and wavelength, polarity, energy, condensation power, and total exposure time.^7^ In addition, exposure can affect the levels of reproductive regulating hormones including FSH, LH, Activin B hormone, inhibitor B, testosterone, and prolactin (PRL).^17^ Inadequate polarization of the cell membrane is responsible for various abnormalities in the process of testosterone synthesis and secretion, which may decrease testosterone levels in the blood and testosterone‐to‐estradiol ratio and decrease spermatogenesis, and eventually lead to infertility.^18^, ^19^ Exposure to EMF can affect the polarization state of the cell membrane. Therefore, damage to the Leydig cells by EMF may lead to lower testosterone levels, thereby reducing the response of Leydig cells to the LH pulse.^20^ The results of human and animal studies have differed on the effects of EMF radiation on reproductive hormones.^21^ While a number of human and animal studies have reported no change in male reproductive hormones at exposure to EMF,^11^, ^30^ others have claimed that exposure to EMF can decrease testosterone levels.^18^, ^20^, ^31^, ^32^, ^33^, ^34^, ^35^ Even in some cases, increases in testosterone, LH, and FSH have been reported.^27^, ^31^, ^33^, ^36^, ^37^

Power plants are workplaces that generate electric and magnetic fields due to the existence of various equipment, such as turbines, generators, internal combustion engines, and cooling towers, which causes staff exposure to these fields and affect their health.^21^, ^38^ Considering the effects of electromagnetic fields on the reproductive system of men and exposure to these fields in power plants, as well as limited human studies in this area and inconsistent results in human and animal studies, the aim of this study is to determine the relationship between exposure rate to fluid density of extremely low‐frequency (ELF) magnetic fields with male reproductive hormone levels.

## 2 ANALYSIS METHOD

### 2.1 Study population

The present cross‐sectional study was a descriptive‐analytical study that was carried out among the employees of different units in the selected power plant around Tehran, Iran. The study population consisted of personnel working in units exposed to extremely low‐frequency magnetic fields. All male participants were asked to complete demographic data sheets, on condition of consent and meeting the inclusion criteria. After selecting participants, GHQ questionnaire and Occupational Exposure Sheet were completed by individuals.

Inclusion criteria included age group of 20‐50 years,^39^ more than 2 years of employment,^40^ no history of infertility in the family,^41^ no history of radiotherapy and chemotherapy,^42^ not taking bodybuilding supplements,^43^ not taking steroid medications (Betamethasone, Dexamethasone, Hydrocortisone, Prednisolone, Nandrolone, Testosterone, etc),^44^ lack of organic diseases affecting sexual function (diabetes, kidney disease, angina pectoris, heart failure, hypertension), lack of testicular swelling and infection, no history of varicocele surgery,^41^ and no history of pituitary gland disease and chronic obstructive pulmonary disease.^44^ All participants were included in the study with informed consent in all stages of implementation. Participation in the study was optional and exclusion criteria included not completing the questionnaire and individual's willingness to leave the study at each stage. At first, demographic information was recorded and in order to comply with ethical considerations, they were assured that their personal information was confidential. The basic principles of the Helsinki Declaration were incorporated in this research project. The study has been approved by the Ethics Committee of the school of Health and Safety of Shahid Beheshti University of Medical Sciences under the ethics code IR.SBMU.PHNS.1389.004.

### 2.2 Measurement of ELF‐MF Exposure

The ELF magnetic field flux density was measured at 135 workstations using TES 1393 calibrated Gauss meter device, Taiwan, by IEEE Std. C95.3.1 method in the study units. The magnetic flux density was measured in μT at a distance of 1 m from the ground (waist area).^45^

Each person was asked about job duties, work place, work duration, a place of rest, and lunch. Then, at all workstations, place of rest and lunch of each individual, field measurements were performed, and the time‐weighted average (TWA) exposure for each individual was calculated using Equation 1.

(1) $$BC=\frac{\sum B\left(t\right)i\times hi}{h}$$

BC—The time‐weighted average of occupational eqxposure to ELF magnetic field (μT)

B(t) _i_—The average magnetic flux density of different parts of each occupational task .(μT)

Hi–Average time spent by the operator for a particular task in different parts of the unit (hour).

h = Duration of a shift (hour).

Employees were divided into different groups based on their exposure levels. The cut‐off point, to divide the exposure groups into three groups of low, moderate, and high exposure, was considered as 33 and 66 percentile. The cut‐off point of the low exposure group (33 per centile) was consistent with the results published by WHO and European countries on exposure to magnetic fields in the home environment.^5^, ^46^, ^47^ Median exposure values in the low, moderate, and high groups were 0.33, 1.51, and 14.78, respectively.

### 2.3 General health questionnaire

Goldberg and Hiller's 28‐item scaled version of the General Health Questionnaire was used to measure the general health of participants. The validity and reliability of the Persian version of this questionnaire are reported by Ebrahimi et al ^48^ at 0.78 and 0.9, respectively, and Cronbach's alpha is 0.97. The questionnaire contains subscales of physical symptoms, anxiety symptoms and sleep disturbances, social functioning, and depression symptoms. This is a 4‐item (0‐3) Likert rating scale questionnaire. In the overall score, a score of 22 or above and a score of 6 on each subscale indicates symptoms.

### 2.4 Measurement of the serum level of reproductive hormones

Between 7‐8 AM, 5cc of blood was taken from the study subjects, by laboratory expert in the power plant dispensary, after 8‐10 h fasting to measure the levels of free testosterone, LH, and FSH.^49^ Blood samples were immediately placed in ice boxes and transferred to the laboratory, and their serum was separated, and stored at −20°C until the collection of all samples.^50^ Serum levels of free testosterone, LH, and FSH were determined by an ELISA method using the StatFax 2100 ELISA reader at 450 nm, according to the kit manufacturer's protocol. Free testosterone was measured by monobind‐96 test kit using the Competitive enzymatic immunological assay, and LH and FSH were measured by PGI‐96 test kit via the sandwich enzymatic immunological assay. Table 1 shows Inter‐ and intra‐assay coefficient of variation (CV) in free testosterone, LH, and FSH.

**Table 1:** Inter‐ and intra‐assay coefficient of variation

Hormone	Inter‐assay CV (%)	Intra‐assay CV (%)
Free testosterone	6.7	9.9
FSH	6.5	4.6
LH	6.5	4.6

Abbreviations: FSH, Follicle stimulating hormone; LH, Luteinizing hormone.

### 2.5 Data analysis

The sample size required for this study was 122 subjects given α = 0.05 and β = 0.2. The normality of quantitative variables was determined by Kolmogorov‐Smirnov test. Descriptive statistics of quantitative variables with normal distribution were reported as mean and standard deviation, with skewed distribution of median and percentile 25 and 75, and for qualitative variables were reported frequency and percentage. Analysis of Variance (ANOVA) and independent t tests were run to compare the mean levels of variables with normal distribution, and Kruskal‐Wallis and Mann‐Whitney tests were used for data with skewed distribution. Correlation tests were employed to investigate the relationship between ELF exposure levels and reproductive hormone levels. We also applied regression analysis for the relationship of hormone levels and ELF while adjusting the smoking status; results were presented as Table 6. We considered linear regression analysis for LH outcome as a continuous variable with normal distribution, and quantile regression analysis was applied for testosterone and FSH variables as continuous variables with non‐normal distribution. Then, we adjusted smoking status in the above‐mentioned models.

All statistical analyses were performed using SPSS software version 25 (Chicago, IL, USA) with 0.05 as the significance level.

## 3 RESULTS

The demographic data and occupational characteristics of individuals are presented in Table 2. Smoking status means current and past use of cigarette, hookah, or pipe. There was no significant statistical difference between the characteristics of the participants in different groups of exposure to an ELF magnetic field.

**Table 2:** Summary of demographic and occupational characteristics of the study subjects

Variable	Mean ± SD /N (%)	ELF magnetic field exposure groups			*P*‐value
		Low	Moderate	High	
Age (year)	37.2 ± 4.3	37 ± 4.5	37.4 ± 4.1	37.2 ± 4.4	.9
Work Experience (year)	13.5 ± 4.8	13.52 ± 5.42	14.1 ± 4.6	13 ± 4.4	.6
Current job work experience (year)	9.9 ± 5.6	9.2 ± 6.1	10.7 ± 5.2	9.9 ± 5.3	.5
BMI **(**kg/m^2^)	26.1 ± 3.2	25.8 ± 2.7	25.7 ± 2.9	26.2 ± 3.9	.3
WHR	0.91 ± 0.06	0.91 ± 0.05	0.9 ± 0.05	0.92 ± 0.07	.3
GHQ	21.9 ± 11.3	23.4 ± 10.9	22.9 ± 12.2	19.65 ± 10.62	.3
Smoking status					
Yes	55 (45.1)	19 (47.5)	17 (41.5)	19 (46.3)	.8
No	66 (54.1)	20 (50)	24 (58/5)	22 (53.7)	
Marital status					
Married	108 (88.1)	35 (87.5)	36 (87.8)	37 (90.2)	.9
Single	13 (10.7)	4 (10)	5 (12.2)	4 (9.8)	

Abbreviations: BMI, Body Mass Index; GHQ, General Health Questionnaire; WHR, Waist‐hip Ratio.

For each individual, the time‐weighted average of exposure to the field was calculated based on the presence at stations with different exposure levels. Employees were divided into low (40 (32.8%)), moderate (41(33.6%)), and high (41(33.6%)) exposure groups, based on their exposure rating. Also hormone levels were reported in different groups of smokers and non‐smokers. Table 3 shows the concentration of free testosterone in different exposure groups, its normal range and percentage of abnormal individuals in the exposure groups. The minimum, maximum, median, and 25 and 75 percentile of free testosterone hormone in the different groups exposure, separately smokers and non‐smokers were reported in Table 3. The level of free testosterone in different ELF exposure groups was compared by Kruskal‐Wallis test. Hormone level in different groups of smokers and non‐smokers was compared by Mann‐Whitney test. There was no significant statistical difference between free testosterone levels in the three exposure groups and in the different groups of smokers and non‐smokers. (*P*‐value > .05).

**Table 3:** Free Testosterone Levels of Participants

Free Testosterone hormone (pg/ml)	Total Participants		Grouping of people by levels of exposure to ELF					
			Low		Moderate		High	
	Smokers	Non‐Smokers	Smokers	Non‐Smokers	Smokers	Non‐Smokers	Smokers	Non‐Smokers
mean ± SD	9.1 ± 4.7	9.2 ± 6.9	9.5 ± 6.2	8.1 ± 3.2	8.8 ± 3.6	8.1 ± 4.5	8.9 ± 3.9	11.3 ± 6.8
Percentile 25	6.1	2.3	5.9	6.6	6.0	6.1	6.2	6.4
Median	7.1	6.9	7.0	6.8	7.1	6.5	7.3	8.1
Percentile 75	11.2	8.6	12.7	7.3	11.6	7.9	10.4	18.3
Minimum	0.8	2.3	0.8	5.4	5.2	2.3	4.8	4.2
Maximum	25.8	25.3	25.8	16.2	16.1	21.4	16.6	25.3
*P*‐value*	−.07		.2					
Normal range	4‐30 pg/ml							
Percentage of abnormal individuals	1.8	1.5	5.2	0	0	4.2	0	0

* Comparison of mean free testosterone level between different exposure groups.

Table 4 shows the concentrations of FSH in different exposure groups, its normal range and the percentage of abnormal individuals in the exposure groups. Also this table presents the minimum, maximum, median, and 25 and 75 percentile of FSH in the three groups, separately smokers and non‐smokers. The level of FSH in different ELF exposure groups was compared by Kruskal‐Wallis test. Hormone level in different groups of smokers and non‐smokers was compared by Mann‐Whitney test. There was no statistically significant difference in FSH levels in the three exposure groups and in the different groups of smokers and non‐smokers. (*P*‐value > .05).

**Table 4:** FSH hormone levels of the participants

Follicle Stimulating (FSH) (mIU/ml)	Total Participants		Grouping of people by levels of exposure to ELF					
			Low		Moderate		High	
	Smokers	Non‐Smokers	Smokers	Non‐Smokers	Smokers	Non‐Smokers	Smokers	Non‐Smokers
mean ± SD	2.8 ± 1.5	3.2 ± 2.1	2.9 ± 1.9	3.1 ± 1.6	2.7 ± 1.5	3.0 ± 2.1	2.7 ± 1.1	3.4 ± 2.4
Percentile 25	1.8	1.9	1.9	2.1	1.4	1.8	1.8	1.3
Median	2.6	2.5	2.6	2.5	2.5	2.4	2.8	2.7
Percentile 75	3.3	4.0	3.3	4.1	3.8	3.2	5.1	5.1
Minimum	0.7	0.8	1.1	1.3	0.8	1.1	0.8	0.8
Maximum	10	10.9	10.0	7.9	6.4	10.9	4.8	9.1
*P*‐value*	−.3		.7					
Normal range	1‐14 mIU/ml							
Percentage of abnormal individuals	3.6	1.5	0	0	5.8	0	5.2	4.5

* Comparison of mean FSH level between different exposure groups.

Table 5 presents the concentration of LH, the minimum, maximum, mean, and standard deviation of LH in the three groups of low, moderate, and high exposure, its normal range, and the percentage of abnormal individuals in different exposure groups. Given the normal distribution of LH, mean and standard deviation were reported. Also, the level of LH was compared through ANOVA test. Hormone level in the different groups of smokers and non‐smokers was compared by Independent *t* test. There was no statistically significant difference between the mean of LH in the three exposure groups and in the different groups of smokers and non‐smokers (*P*‐value > .05).

**Table 5:** LH hormone levels of participants

Follicle Stimulating (FSH) (mIU/ml)	Total Participants		Grouping of people by levels of exposure to ELF					
			Low		Moderate		High	
	Smokers	Non‐Smokers	Smokers	Non‐Smokers	Smokers	Non‐Smokers	Smokers	Non‐Smokers
Mean ± SD	2.5 ± 1.1	2.7 ± 1.4	2.3 ± 1.1	2.5 ± 1.1	2.3 ± 0.9	2.8 ± 1.7	2.9 ± 1.1	2.7 ± 1.3
Minimum	0.4	0.6	0.4	1.0	1.5	0.6	1.1	0.7
Maximum	4.8	6.7	4.7	6.4	4.7	6.7	4.8	5.5
*P*‐value*	.1		.4					
Normal range	0.7‐7.4 mIU/ml							
Percentage of abnormal individuals	1.5	1.8	5.2	0	0	4.2	0	0

* Comparison of mean LH level between different exposure groups.

**Figure 1:**
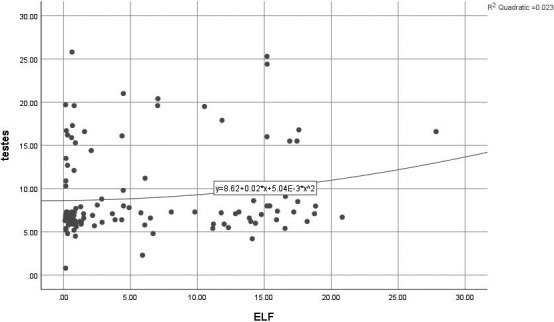
Scatter plot of relationship between serum level of free testosterone and exposure to ELF‐TWA magnetic field

The relationship between serum‐free testosterone level and ELF magnetic field flux was examined using the Spearman correlation coefficient test, and no significant relationship was observed (*P* = .08, *r* = .15) (Fig 1).

**Figure 2:**
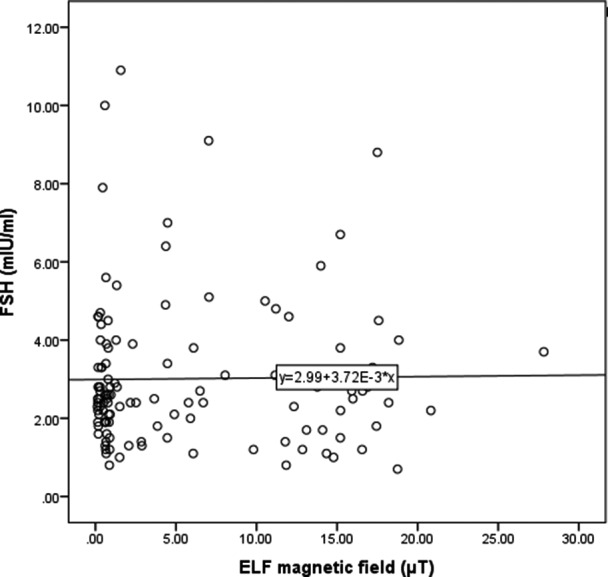
Scatter plot of relationship between serum level of FSH and exposure to ELF‐TWA magnetic field

**Figure 3:**
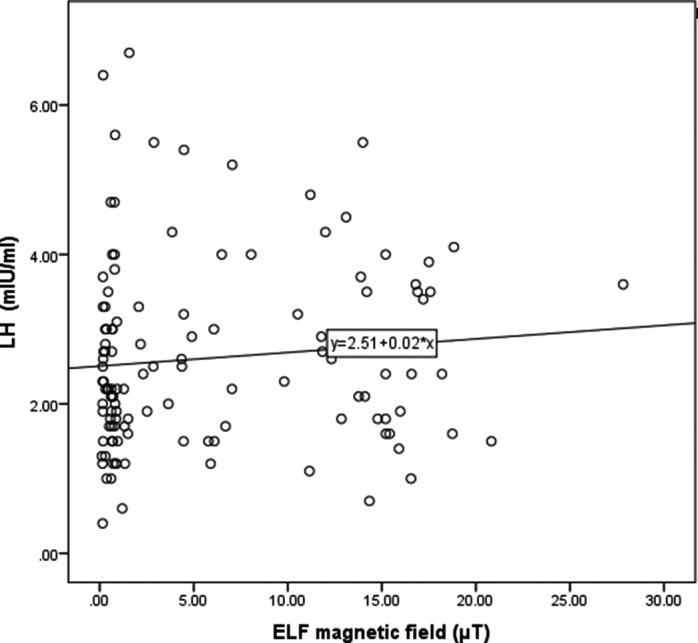
Scatter plot of relationship between serum level of LH and exposure to ELF‐TWA magnetic field

The correlation between serum levels of LH and FSH by mean weighted time of exposure to an ELF magnetic field is shown in Figures 2 and 3, indicating no statistically significant relationship between these hormones and ELF magnetic field (*P* = .28), (*P* = .88) (Figures 2 and 3).

Investigating the relationship between underlying factors and reproductive hormone levels, we found a significant relationship among age, general work experience, and LH. With age, LH decreased by 0.25 per year and as work experience increased, it declined by 0.20 per year (*P*‐value = .025, *P*‐value = .005). With age and overall work experience, LH levels decreased. There was no statistically significant relationship between the baseline variables and free testosterone and FSH.

The results of regression analysis for the relationship of hormone levels and ELF adjusted for smoking status are shown in Table 6. There was no significant relationship between ELF with LH and FSH, even with modulating the effect of smoking. However, by modulating the effect of smoking, a weak correlation was observed between ELF and free testosterone, which increased the testosterone level by 0.05 per unit increase to ELF.

**Table 6:** Relationship between hormone levels and ELF for smoking status

Models	Linear regression				Quantile regression							
Hormone	LH				FSH				Testosterone			
Variables	Coef.	SE	95% CI	*P*‐value	Coef.	SE	95% CI	*P*‐value	Coef.	SE	95% CI	*P*‐value
ELF	0.02	0.02	(−0.01,0.05)	.3	0.01	0.01	(−0.02,0.04)	.4	0.05	0.02	(0.01,0.1)	.08
Smoker (yes)	−0.2	0.2	(−0.6, 0.3)	.4	0.1	0.2	(−0.3, 0.5)	.6	0.02	0.3	(−0.5,0.6)	.9

## 4 DISCUSSION

The results showed that the intensity of ELF magnetic field in all measurement stations was below the Occupational Exposure Limit (OEL) recommended by the Iranian Occupational Exposure Review and Adjustment Limit and Threshold Limit Value (TLV) recommended by the American Conference of Governmental Industrial Hygienists (ACGIH) (Limit Value of ELF, 0.2‐60 mT).^51^, ^52^ The results of this study are consistent with the study of Alizadeh et al and Bagheri Hossein Abadi et al^38^, ^53^, ^54^ In several other studies, the ELF magnetic field rate in the power plant was lower than the permitted level.^21^, ^37^ After preparation and reading of the kit using ELISA Reader, serum testosterone, LH, and FSH concentrations of the blood of participants were 0.8‐25.8 pg/ml, 0.4‐6.7 mIU/ml, and 0.7‐10.9 mIU/ml, respectively. With respect to the normal range, 98.4%, 98.4%, and 97.5% of subjects were in normal range.

This study showed that there was no relationship between exposure to ELF magnetic field and reproductive hormones in men without a history of infertility, who worked in the power plant.

Exposure to these fields can cause atrophy in seminal tubes, decrease testosterone levels, and hyperplasia of Leydig cells in a compensatory reaction to lower testosterone concentration. This increase in Leydig cells may compensate for the decrease in total volume occupied by Leydig cells and keep plasma testosterone levels at normal levels. Thus, it seems that the effect of these waves on testosterone levels was not due to central inhibition of pituitary hormone secretion, but rather to the peripheral environment.^29^, ^30^, ^33^

There have been numerous human and animal studies on the effect of the exposure to magnetic fields on fertility, with contradictory results.^21^, ^22^, ^24^, ^31^, ^55^

In the study by Hjollund et al, no significant relationship was found between ELF exposure of men and women and human reproductive markers. The results of this study, as in the present study, did not show any adverse effect of exposure to an ELF magnetic field on fertility markers.^24^

In the study of men with backache who were treated with magnet therapy, the effects of chronic exposure to ELF magnetic fields on FSH, LH, prolactin, testosterone, and estradiol concentrations were investigated. One month after magnet therapy, LH levels decreased significantly compared with baseline, whereas a slight increase was observed after magnetic stimulation. LH levels decreased significantly 1 month after magnet therapy compared with baseline, whereas a slight increase was observed after magnetic stimulation. No statistically significant changes were observed in FSH and testosterone levels after either treatment at either time point. Chronic exposure to magnetic field has been reported as the cause of LH depletion in this study.^25^

Numerous animal studies have been performed to investigate the effect of electromagnetic fields on reproductive hormones.

The results of a study by Mustafa et al showed that a magnetic field of 5 mT for 1, 2, or 4 weeks had no significant effect on serum testosterone levels in male rats. FSH level in the exposure group for 1 week and LH level in the exposure group for 4 weeks showed a significant increase compared to the control group. In this study, a significant increase in FSH levels along with a slight decrease in testosterone levels may be due to the effect of magnetic field on the germinal epithelium with sufficient compensatory increase in gonadotropin level to maintain the normal quality of semen.^27^ Exposure to the magnetic field of power lines in male rats did not cause significant changes in the number of children, days of pregnancy, sperm count, and testosterone level in the exposed group compared to the control group.^22^ The level of magnetic field exposure in this study was similar to the present study, and similar results were obtained.

In several animal studies, as in the present study, no significant relationship was observed between exposure to the magnetic field and reproductive hormones.^11^, ^28^, ^29^ In the study of Al‐Akhras et al (2006), mice exposed to 25 μT for 18 weeks had significantly reduced levels of testosterone after 6 and 12 weeks of exposure, but changes in testosterone levels after 18 weeks were not significant. LH levels increased after 18 weeks of exposure. No effect was observed on FSH.^31^

Contrary to the results of the present study, Wang et al's study showed a negative relationship between occupational EMF exposure and plasma testosterone. The results showed that chronic exposure to EMF can decrease testosterone levels in men. In this study, subjects in the high‐exposure group were exposed to Walkie Talkie radio RF‐EMF as well as ELF‐EMF. There was also a significant difference in work experience between the two groups.^21^

Gamberale et al investigated the possible acute effects of exposure to ELF electric and magnetic fields on reproductive hormones at three different time points under exposure and control conditions in linemen. Results showed significant difference only in testosterone in control and exposure conditions, which was higher in exposure conditions, with the results being in contradiction with those of the present study. The observed difference in testosterone concentration appears to be due to the effect of work‐related physical load and the daily variance of this hormone concentration. The LH and FSH hormones did not change significantly.^37^ In the present study, blood samples were taken according to the daily variation in reproductive hormones only at one time point at 7‐8 AM, that is, the hormone levels are at the highest level in the circadian rhythm changes,^56^ to eliminate the effects of the daily variance of these hormones. In this study, hormones were studied at three different time points in the shift, and the effect of daily variance of testosterone on the results was evident.

Other reasons for these results are the low sample size in different exposure groups and the relatively low difference in exposure levels of the ELF magnetic fields in different exposure groups; the variance of the hormones was studied in the subjects.

The results of a study showed the effect of ELF‐EMF at 1 Hz and 50 Hz on spermatogonial proliferation, and differentiation in mice and increase in meiotic division, whole sex cell division, and serum testosterone concentration were observed.^36^

In three animal studies in which exposure to the magnetic field was 1 ms, different results were obtained. In one study, plasma levels of testosterone remained unchanged,^30^ and in the others, plasma levels of testosterone decreased.^32^, ^33^ The frequency of the magnetic field and the duration of exposure were different in these studies. In the study of Gholampour et al (2012), atrophy in seminiferous and larger interstitial tissue such as Leydig cell hyperplasia was observed in the exposure group after 135 days. Testosterone is essential in high quantity to maintain the function of the reproductive system. Therefore, atrophy in seminiferous may decrease the testosterone levels observed. Leydig cell hyperplasia may also be a compensatory response to lower testosterone concentrations.^33^ In a study by Tenorio et al, it was reported that an increase in the level of Leydig cells observed may compensate for the decrease in the total volume occupied by Leydig cells, which maintains plasma testosterone levels at normal levels.^30^

The results of some studies indicate a decrease in reproductive hormones at the exposure of ELF magnetic field but some have not seen any relationship. Despite ample research on the interaction of EMF with biological systems, various aspects are still unclear, and the reported results are controversial. The difference observed in the results of studies regarding the potential toxicity of electric and magnetic fields can be related to differences in the frequency or intensity of the field, exposure protocol, species and race of animals, and differences in exposure time.^57^ The precise mechanism of how ELF‐EMFs may affect serum testosterone levels has not yet been elucidated. A number of studies on mice exposed to ELF‐EMF have reported higher serum LH levels along with a decrease in testosterone levels.^31^, ^55^ This suggests that the effect of ELF‐EMF on testosterone levels is not due to central inhibition of pituitary hormone secretion, but rather it might be caused by a peripheral inhibition.^29^

The particular challenge in evaluating EMF exposure is that it is ubiquitous and it is difficult to find an unexposed control group. For this reason, there is little contrast between comparing low exposure versus high exposure levels. Other challenges in research on the effects of EMF on health are the lack of awareness of the mechanism of biological and biophysical action of EMF at peripheral exposure level.^58^

Many EMF‐related diseases are chronic and require retrospective studies, which in turn makes it more difficult to assess exposure.

In many studies in this field, the number of people exposed to high magnetic field is low, which makes it impossible to arrive at a definitive conclusion about the effect of high level of magnetic field exposure. Using self‐reported exposure data will bias the results.^59^

Limitations of this study include lack of proper evaluation of fertility status, failure to use preferred testosterone hormone measurement method, one‐time measurement limit, no significant difference in exposure levels between individuals and lack of evaluation of exposure to other sources.

## 5 CONCLUSION

According to the results of this study, there was no significant relationship between exposure to ELF and levels of reproductive hormones (free testosterone, LH, and FSH). Therefore, at this level of exposure to the ELF magnetic field, there is no significant relationship between exposure and reproductive hormones of free testosterone, LH, and FSH in men working in the plant.

Despite the results of the present study, given that a number of studies indicate the potential adverse effects of low‐frequency electromagnetic fields on reproductive markers, there is a need for further research in order to reach more conclusive and convincing results.

To conclude with greater certainty about the effect of the magnetic field on reproductive hormones, it is advisable to conduct studies with a more precise exposure assessment and use of sufficient sample volume, and applying various levels of exposure to the magnetic field. Also, a simultaneous study of reproductive biological biomarkers with greater sensitivity to the magnetic field is needed. It seems that the level of reproductive hormones is not a good indicator for evaluating the performance of the reproductive system because of the extent of the natural range of these hormones. More accountable results would be achieved if the spermatogenesis index were investigated in this study.
